# R3Design: deep tertiary structure-based RNA sequence design and beyond

**DOI:** 10.1093/bib/bbae682

**Published:** 2024-12-31

**Authors:** Cheng Tan, Yijie Zhang, Zhangyang Gao, Hanqun Cao, Siyuan Li, Siqi Ma, Mathieu Blanchette, Stan Z Li

**Affiliations:** Zhejiang University, Zhejiang, China; AI Lab, Research Center for Industries of the Future, Westlake University, Zhejiang 310058, China; School of Computer Science, McGill University, Montreal QC H3A 2T8, Canada; MILA - Québec AI Institute, Montreal QC H2S 3H1, Canada; AI Lab, Research Center for Industries of the Future, Westlake University, Zhejiang 310058, China; Department of Computer Science and Engineering, The Chinese University of Hong Kong, Shatin, N.T., Hong Kong, China; AI Lab, Research Center for Industries of the Future, Westlake University, Zhejiang 310058, China; AI Lab, Research Center for Industries of the Future, Westlake University, Zhejiang 310058, China; School of Computer Science, McGill University, Montreal QC H3A 2T8, Canada; MILA - Québec AI Institute, Montreal QC H2S 3H1, Canada; AI Lab, Research Center for Industries of the Future, Westlake University, Zhejiang 310058, China

**Keywords:** RNA, artificial intelligence, inverse folding, graph neural networks, biomolecular engineering

## Abstract

The rational design of Ribonucleic acid (RNA) molecules is crucial for advancing therapeutic applications, synthetic biology, and understanding the fundamental principles of life. Traditional RNA design methods have predominantly focused on secondary structure-based sequence design, often neglecting the intricate and essential tertiary interactions. We introduce R3Design, a tertiary structure-based RNA sequence design method that shifts the paradigm to prioritize tertiary structure in the RNA sequence design. R3Design significantly enhances sequence design on native RNA backbones, achieving high sequence recovery and Macro-F1 score, and outperforming traditional secondary structure-based approaches by substantial margins. We demonstrate that R3Design can design RNA sequences that fold into the desired tertiary structures by validating these predictions using advanced structure prediction models. This method, which is available through standalone software, provides a comprehensive toolkit for designing, folding, and evaluating RNA at the tertiary level. Our findings demonstrate R3Design’s superior capability in designing RNA sequences, which achieves around $44\%$ in terms of both recovery score and Macro-F1 score in multiple datasets. This not only denotes the accuracy and fairness of the model but also underscores its potential to drive forward the development of innovative RNA-based therapeutics and to deepen our understanding of RNA biology.

## Introduction

The pivotal role of Ribonucleic acid (RNA) in biological systems is underscored by its diverse functions, from encoding genetic information to catalyzing biochemical reactions and regulating gene expression [1–3]. Notably, non-coding RNA strands fold into complex three-dimensional structures that are crucial for their biological functionality [4, 5]. The design of RNA molecules with specific structures and functions has profound implications for therapeutic development, synthetic biology, and the elucidation of life’s molecular underpinnings [6, 7]. The intricate geometries intrinsic to RNA molecules equip them with unique capabilities [8], enabling them to perform irreplaceable roles in vital cellular operations, including but not limited to mRNA translation [9], RNA splicing [10–12], and gene regulation [13]. These processes are foundational to cellular biology, underscore the critical nature of RNA’s contributions to life sciences, and highlight the potential impact of mastering RNA design on future biomedical and biotechnological advancements.

Despite the crucial role of RNA in myriad biological processes, the ability to design RNA molecules that fold into specific three-dimensional structures with high precision remains a significant challenge. Traditional computational methods for RNA design have predominantly focused on secondary structure predictions [14–19]. Some more recent approaches not only focused on developing reinforcement learning tools to enhance secondary structure-based RNA sequence design [14], but also proposed a standard protocol to integrate a 3D structure prediction model with the system pipeline to promote a more realistic sequence design [20], which accepts the designed sequence depending on the quality of predicted 3D structures with that sequence. However, while those methods are important and inspiring, their reliance on RNA secondary structure offers an incomplete view of RNA’s functional capabilities [21–25]. In particular, algorithms for RNA secondary structure prediction have been extensively developed, yielding impressive results through leveraging large datasets of known secondary structures [26–29]. However, knowledge of RNA tertiary structures, which is crucial for thoroughly understanding RNA functional mechanisms and discovering RNA-targeted therapies [6, 30], remains limited [31].

The success of protein structure prediction approaches [32, 33] inspired similar advancements in RNA tertiary structure prediction, leading to the development of RNA tertiary structure folding algorithms such as DeepFoldRNA [34], RoseTTAFoldNA [35], DRfold [25], trRosettaRNA [24], and RhoFold [36, 37]. While predicting RNA tertiary structures from primary sequences can leverage abundant sequence data [36] by multiple sequence alignment or language models, its inverse problem, designing RNA sequences that reliably fold into a specified tertiary structure, as also proposed by recent approaches [38], remains largely underexplored.

In this work, we propose a thorough pipeline aiming at data-driven tertiary structure-based RNA design tasks. We introduce R3Design, an RNA sequence design method that trained on over two thousand representative RNA structures collected from Protein Data Bank (PDB) [39] and RNASolo dataset [40]. R3Design builds a computational framework tailored to tackle the complexity of RNA tertiary structures. Furthermore, R3Design incorporates base pair prediction prior to guiding the RNA design process, leveraging the correlation between RNA secondary and tertiary structures. An iterative refinement strategy fine-tunes the model’s outputs through cycles of prediction and adjustment, facilitating nuanced adjustments that align with the complex structural dynamics inherent to functional RNA molecules. Benchmark evaluations underscore the efficacy of R3Design, establishing a robust baseline for tertiary structure-based RNA design and paving the way for future innovations in RNA-based therapeutics and molecular biology.

## Results

### R3Design designs RNA sequences with high sequence-level fidelity

We collected a non-redundant set of RNA structures from the PDB [39] and RNASolo dataset [40] to train R3Design, which comprises a total of 2218 representative RNA structures. This dataset was initially derived from the representative RNA structures in the RNASolo dataset with a resolution less than 4.0 Å. Then, their sequences and structures were cleaned according to the corresponding structures in the PDB database. Specifically, we excluded the sequences longer than 500 nucleotides as they only occupy $4.21\%$ in the whole original dataset, as shown in the Appendix. Our dataset was curated to represent a broad range of RNA structural types and complexities, ensuring a robust test of R3Design’s capabilities. We divided the dataset based on structural similarity, allocating 1774 for training, 223 for validation, and 221 for testing purposes. The distribution and specific characteristics of this dataset are detailed in the Appendix. To ensure the reliability of our results, we conducted each experiment three times using different random seeds and reported both the mean and standard deviation of our metrics, providing insights into the consistency and precision of R3Design.

Table 1 presents the sequence recovery rates achieved by R3Design in comparison to established RNA design methods across three sequence length categories: Short (0–50 nucleotides), Medium (50–100 nucleotides) and Long (more than 100 nucleotides). The recovery rate measures the percentage of nucleotides in the designed sequence that exactly match the target sequence, providing a direct indicator of fidelity. Higher values indicate better performance. Notably, R3Design demonstrates superior performance, particularly highlighted by its consistency across varying complexities and lengths, which underscores its robustness in handling the intrinsic variability of RNA structures.

**Table 1:** The recovery on the benchmark dataset. The best results are highlighted in bold.

Method	Recovery (%) $\uparrow $			
	Short	Medium	Long	All
SeqRNN (h=128)	26.52$\pm $1.07	24.86$\pm $0.82	27.31$\pm $0.41	26.23$\pm $0.87
SeqRNN (h=256)	27.61$\pm $1.85	27.16$\pm $0.63	28.71$\pm $0.14	28.24$\pm $0.46
SeqLSTM (h=128)	23.48$\pm $1.07	26.32$\pm $0.05	26.78$\pm $1.12	24.70$\pm $0.64
SeqLSTM (h=256)	25.00$\pm $0.00	26.89$\pm $0.35	28.55$\pm $0.13	26.93$\pm $0.93
StructMLP	25.72$\pm $0.51	25.03$\pm $1.39	25.38$\pm $1.89	25.35$\pm $0.25
StructGNN	27.55$\pm $0.94	28.78$\pm $0.87	28.23$\pm $1.95	28.23$\pm $0.71
GraphTrans	26.15$\pm $0.93	23.78$\pm $1.11	23.80$\pm $1.69	24.73$\pm $0.93
PiFold	24.81$\pm $2.01	25.90$\pm $1.56	23.55$\pm $4.13	24.48$\pm $1.13
R3Design	**39.66** $\pm $ 2.30	**47.04** $\pm $ 0.39	**47.42** $\pm $ 0.93	**44.27** $\pm $ 0.62

The Macro F1-score, presented in Table 2, evaluates the balance between precision and recall achieved by each method across different RNA sequence lengths. The score is multiplied by 100 for better readability. A higher Macro F1-score indicates a method’s efficiency in not only identifying correct nucleotides (precision) but also in minimizing false negatives (recall). R3Design’s consistently higher scores across all categories reflect its robustness in sequence prediction, substantially enhancing both aspects of prediction quality compared to other methods.

**Table 2:** The Macro-F1 on the benchmark dataset. The score is multiplied by 100 for aesthetics.

Method	Macro F1 ($\times $100) $\uparrow $			
	Short	Medium	Long	All
SeqRNN (h=128)	17.22$\pm $1.69	17.20$\pm $1.91	8.44$\pm $2.70	17.74$\pm $1.59
SeqRNN (h=256)	12.54$\pm $2.94	13.64$\pm $5.24	8.85$\pm $2.41	13.64$\pm $2.69
SeqLSTM (h=128)	9.89$\pm $0.57	10.44$\pm $1.42	10.71$\pm $2.53	10.28$\pm $0.61
SeqLSTM (h=256)	9.26$\pm $1.16	9.48$\pm $0.74	7.14$\pm $0.00	10.93$\pm $0.15
StructMLP	17.46$\pm $2.39	18.57$\pm $3.45	17.53$\pm $8.43	18.88$\pm $2.50
StructGNN	24.01$\pm $3.62	22.15$\pm $4.67	26.05$\pm $6.43	24.87$\pm $1.65
GraphTrans	16.34$\pm $2.67	16.39$\pm $4.74	18.67$\pm $7.16	17.18$\pm $3.81
PiFold	17.48$\pm $2.24	18.10$\pm $6.76	14.06$\pm $3.53	17.45$\pm $1.33
R3Design	**41.48** $\pm $ 0.32	**45.16** $\pm $ 2.28	**42.80** $\pm $ 3.65	**44.44** $\pm $ 0.85

To test the generalizability of R3Design, we further evaluated its performance on external benchmark datasets, including Rfam and RNA-Puzzles, which were compiled in [34]. These datasets encompass a diverse array of RNA structures and complexities, providing a robust framework for evaluating R3Design. We pre-trained the R3Design model on our benchmark dataset, explicitly excluding RNA structures that were similar to those in the external datasets to prevent data leakage and ensure a stringent testing protocol. Subsequently, we assessed the model’s performance on these benchmarks directly, without any additional training or fine-tuning.

The results, presented in Table 3, demonstrate that R3Design not only adapts well to new RNA structures but also consistently outperforms all baseline methods in terms of recovery and Macro F1-scores. R3Design achieved the highest recovery scores of 43.27% on Rfam and 45.41% on RNA-Puzzles, significantly outperforming the nearest competitor, eM2dRNAs, which scored 33.34 and 37.10%, respectively. This metric directly reflects the ability of R3Design to accurately reproduce target RNA sequences from their tertiary structures. Similarly, R3Design’s Macro F1 scores were 41.37% on Rfam and 44.74% on RNA-Puzzles, substantially higher than those of all other methods. The closest scores were by eM2dRNAs, at 24.80% on Rfam and 26.91% on RNA-Puzzles. These results highlight R3Design’s ability to generalize from its training dataset to new, previously unseen RNA structures. These findings affirm R3Design’s robust capability to model RNA sequences with high fidelity across varying structural complexities and datasets. The notable improvements in sequence recovery and Macro F1 scores underline not only its precision but also its reliability and effectiveness.

**Table 3:** The overall recovery and Macro-F1 scores on the Rfam and RNA-Puzzles datasets.

Method	Recovery (%) $\uparrow $		Macro F1 ($\times $100) $\uparrow $	
	Rfam	RNA-Puzzles	Rfam	RNA-Puzzles
SeqRNN (h=128)	31.05$\pm $0.51	31.51$\pm $0.05	11.92$\pm $0.17	12.11$\pm $0.03
SeqRNN (h=256)	31.04$\pm $0.50	31.53$\pm $0.04	11.93$\pm $0.16	12.12$\pm $0.02
SeqLSTM (h=128)	30.28$\pm $0.20	31.35$\pm $0.26	12.36$\pm $0.15	12.40$\pm $0.15
SeqLSTM (h=256)	31.45$\pm $0.08	31.79$\pm $0.44	11.76$\pm $0.09	12.07$\pm $0.00
StructMLP	26.77$\pm $3.38	27.06$\pm $3.81	16.22$\pm $2.43	16.72$\pm $2.53
StructGNN	20.81$\pm $1.42	20.68$\pm $0.70	14.54$\pm $1.11	12.70$\pm $2.60
GraphTrans	27.50$\pm $4.15	25.69$\pm $4.34	20.66$\pm $2.51	20.17$\pm $0.14
PiFold	22.55$\pm $4.13	23.78$\pm $6.52	16.08$\pm $2.34	16.20$\pm $3.49
MCTS-RNA	31.74$\pm $0.07	32.06$\pm $1.87	23.82$\pm $4.60	24.12$\pm $3.47
LEARNA	31.92$\pm $2.37	30.94$\pm $4.15	24.02$\pm $3.73	22.75$\pm $1.17
aRNAque	30.01$\pm $3.26	31.07$\pm $2.32	22.84$\pm $1.70	23.30$\pm $1.65
eM2dRNAs	33.34$\pm $1.02	37.10$\pm $3.24	24.80$\pm $3.88	26.91$\pm $2.32
R3Design	**43.27** $\pm $ 0.56	**45.41** $\pm $ 1.95	**41.37** $\pm $ 1.27	**44.74** $\pm $ 0.71

**Figure 1:**
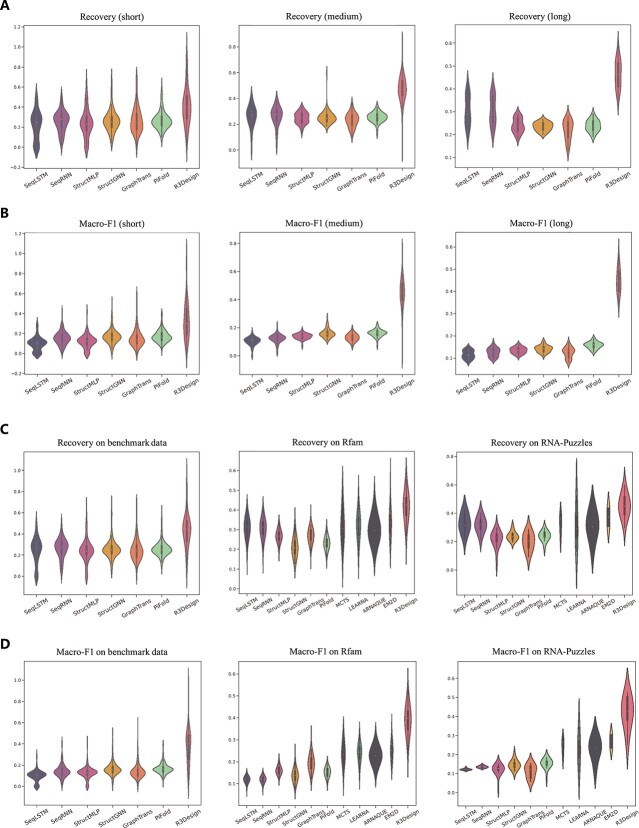
Violin plot on the sequence-level metrics across our benchmark, Rfam, and RNA-Puzzles datasets. (A) The first row shows the recovery rate comparison on the benchmark dataset with short, medium, and long splits. (B) The second row shows the Macro F1 comparison on the benchmark dataset with short, medium, and long splits. (C) The third row shows the recovery rate comparison on the complete benchmark dataset, Rfam, and RNA-Puzzles datasets. (D) The fourth row shows the Macro F1 comparison on the complete benchmark dataset, Rfam, and RNA-Puzzles datasets.

For better visualization and to provide a clearer comparison of the distribution of the sequence-level metrics, we present a violin plot in Fig. 1. These results underscore R3Design’s capability to accurately model RNA sequences with high fidelity across different structural complexities and datasets. The improvements in sequence recovery and Macro-F1 scores not only highlight its precision but also its reliability and effectiveness for practical applications in RNA-based therapeutics.

### The designed sequence can fold into desirable secondary structure

Achieving high sequence-level fidelity is crucial for the accurate synthesis of RNA molecules; however, the functional competence of these molecules also critically depends on their ability to adopt correct secondary structures. This ability is pivotal not only for the structural integrity of RNA but also for its functionality in biological processes such as catalysis, regulation, and interactions with other biomolecules. To evaluate R3Design’s efficacy, we investigate its capacity to ensure that sequences it designs accurately fold into their native secondary structures. For our analyses, secondary structures are represented using the dot-bracket notation, which provides a visual and statistical means to assess folding accuracy.

Specifically, we leverage ModeRNA [41] to convert the tertiary structures of each RNA molecule into their corresponding dot-bracket representations. These representations served as the ground truth in our subsequent analyses. For the RNA sequences designed by R3Design, we employed RNAfold, a component of the ViennaRNA [42], to predict their secondary structures. RNAfold is renowned for its accuracy and efficiency in determining RNA secondary structures from sequence data, using thermodynamically optimized algorithms to predict the most stable structural configuration under given conditions. By inputting the designed sequences, we can obtain a predicted secondary structure in the dot-bracket notation for each sequence, which we then compare against the ground truth. We use accuracy to measure the percentage of nucleotides in the designed RNA sequence that correctly matches the secondary structure predicted by RNAfold. A higher percentage indicates a more accurate prediction of the RNA’s secondary structure. It is important to note that RNAfold may not always successfully predict a secondary structure for certain sequences. Therefore, we also calculated the ’recovered sequence rate’, which reflects the percentage of designed sequences that RNAfold could successfully fold into secondary structures. A rate of 100% indicates that all input sequences were successfully predicted.

**Figure 2:**
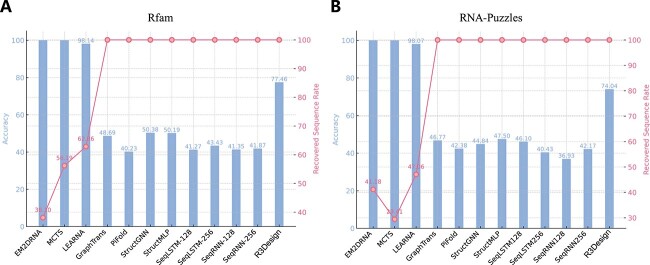
Performance comparison of R3Design and other baseline models on folding secondary structure. The accuracy is the predicted secondary structure based on the designed sequences, and the recovered sequence rate is the foldable sequence rate. (A) The metrics on the Rfam dataset. (B) The metrics on the RNA-Puzzles dataset.

We summarize the performance in designing RNA sequences that correctly fold into their predicted secondary structures in Fig. 2. On the Rfam dataset, R3Design stands out with an accuracy of 77.46%, which is significantly higher than the other methods, all while maintaining a 100% recovered sequence rate. Methods like MCTS, LEARNA, and EM2DRNA show perfect accuracy but their usability is limited by lower recovered sequence rates, suggesting that while they are highly precise, they are not as versatile or generalizable. Similar to the Rfam dataset, R3Design again provides a superior balance of high accuracy (74.04%) combined with a 100% recovered sequence rate on the RNA-Puzzles dataset, affirming its effectiveness and reliability in RNA sequence design across diverse datasets.

### The designed sequence can fold into desirable tertiary structure

We evaluate the capability of R3Design to design RNA sequences that accurately fold into their desired tertiary conformations, using several advanced RNA tertiary structure prediction models for validation. To assess the tertiary folding of RNA sequences designed by R3Design, we employed three prominent RNA tertiary structure prediction models: DRfold [25], trRosettaRNA [24], AlphaFold3 [43], and RoseTTAFoldNA [35]. For each model, we first predicted the structures of native sequences as a baseline and then applied the models to sequences designed by R3Design. The Root Mean Square Deviation (RMSD) from the ground-truth structures served as the primary metric for evaluating the folded RNA tertiary structures.

**Figure 3:**
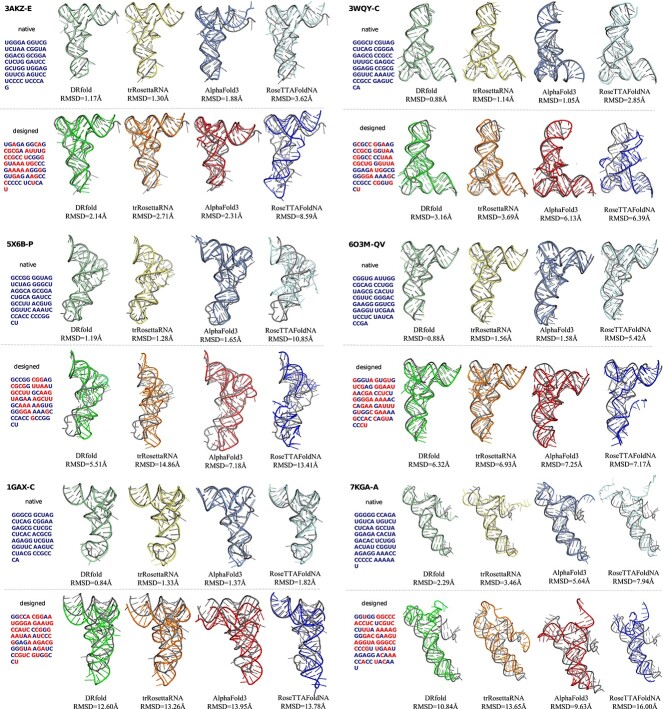
Comparative analysis of tertiary structure predictions for RNA sequences designed by R3Design. For each RNA molecule, we display both the native sequence and the sequence designed by R3Design. Tertiary structures predicted from these sequences using DRfold, trRosettaRNA, AlphaFold3, and RoseTTAFoldNA are shown. RMSD values are calculated to assess the accuracy of the predicted structures relative to the actual native tertiary structures. We highlight the different bases in the designed sequences in red.

We selected a set of RNA molecules that were unseen during the training of R3Design and are representative of diverse RNA structural types and complexities. Each molecule’s native sequence, along with the sequence redesigned by R3Design, was analyzed to compare their ability to fold into tertiary structures as predicted by the aforementioned models (Fig. 3).

**Low RMSD cases (3AKZ-E and 3WQY-C)**

For the RNA molecules 3AKZ-E and 3WQY-C, despite the designed sequences being markedly different from their native counterparts, the tertiary structures predicted by DRfold and trRosettaRNA closely resembled the native structures, as indicated by their low RMSD values. This suggests that R3Design can effectively design functionally equivalent RNA structures, even with significant sequence changes, maintaining structural integrity as evaluated by these models.

**Moderate RMSD cases (5X6B-P and 6O3M-QV)**

In cases such as 5X6B-P and 6O3M-QV, although the tertiary structures predicted from the R3Design sequences appeared visually similar to the ground-truth structures, they exhibited relatively higher RMSD values. Notably, DRfold consistently outperformed other models, indicating the combination of DRfold and R3Design is promising in in-silico tertiary structure-based RNA sequence design.

**High RMSD cases (1GAX-C and 7KGA-A)**

For molecules like 1GAX-C and 7KGA-A, the predicted structures displayed higher RMSD values yet maintained a similarity to the native structures in terms of overall spatial conformation. These instances underscore the challenges in RNA design, particularly in cases where maintaining exact native-like structures is crucial. Despite these challenges, the results affirm that R3Design possesses the capability to design RNA sequences that generally fold into their desired tertiary structures with high structural fidelity.

### An RNA tertiary structure-based design software for in-silico designing and screening

The R3Design software represents a computational platform specifically tailored for the in-silico design and analysis of RNA sequences. By leveraging the inherent tertiary structure data provided by input PDB files, this software enables the RNA sequence design to meet desired structures. This innovative tool integrates sequence redesign, and comprehensive structural evaluations, making it a useful resource for researchers in the field of synthetic biology and therapeutic development.

**Figure 4:**
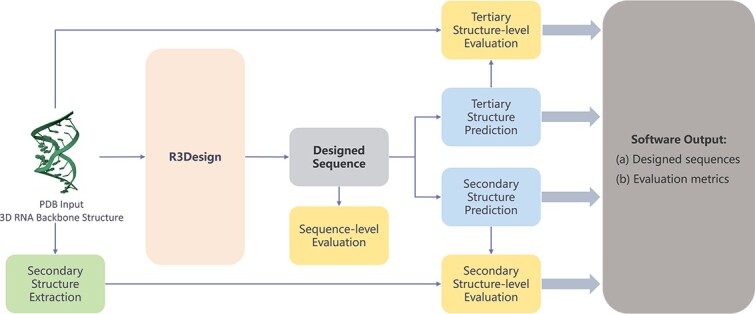
The detailed modular architecture of the R3Design software. The pipeline comprises three main components: (i) RNA sequence redesign using R3Design, based on the input tertiary structure, (ii) comprehensive evaluations at the sequence level, secondary structure level, and tertiary structure level, and (iii) the final output, which includes the optimized RNA sequences along with their corresponding evaluation metrics.

As illustrated in Fig.4, the R3Design web software orchestrates the in-silico RNA design process through a streamlined, three-component pipeline: RNA sequence design using R3Design, comprehensive evaluations, and the delivery of final outputs. This platform begins by ingesting an RNA’s tertiary structure through a PDB file, setting the stage for RNA sequence design. We first utilize the R3Design model to design RNA sequences based on input tertiary structures. Following the sequence design, the sequences undergo meticulous evaluations across three structural dimensions—sequence, secondary, and tertiary levels:

- Sequence-level evaluation: this initial assessment focuses on sequence integrity, employing metrics like the recovery rate and Macro F1-score to quantify the similarity between the designed and native sequences.
- Secondary structure-level evaluation: the accuracy of the predicted secondary structures is then verified, which is essential for understanding the RNA’s structural feasibility and the likelihood of it achieving the correct fold.
- Tertiary structure-level evaluation: we compare the predicted structures, obtained using advanced models like RoseTTAFoldNA, with the original tertiary structures from the PDB input. This comparison is crucial as it highlights the structural fidelity and functional viability of the designed RNA sequences, offering insights into the effectiveness of the R3Design modifications.

The software outputs the designed RNA sequences that are capable of folding into the desired tertiary structures. Accompanying the designed sequences are detailed evaluation metrics that provide insights into the structural accuracy at multiple levels (tertiary, secondary, and sequence). Each metric serves as a critical component of the RNA sequence design process, elucidating successes and pinpointing areas that may require further optimization.

This R3Design software marks a significant advancement in tertiary structure-based RNA sequence design, offering a powerful platform for the in-silico design and evaluation of RNA molecules. By systematically redesigning RNA sequences based on the given tertiary structures and providing exhaustive multi-level structural evaluations, this software aids researchers in synthesizing RNA molecules with enhanced properties and confirmed structural integrity. Its comprehensive output, including optimized sequences and detailed metrics, ensures that researchers are well-equipped to pursue further experimental validations and applications in synthetic biology.

## Discussion

We developed R3Design, a tertiary structure-based RNA sequence design model, diverging from the traditional secondary structure-based models commonly used in RNA design. We evaluate R3Design across three critical aspects to ensure its effectiveness and superiority over existing methods: sequence-level fidelity, secondary structure folding, and tertiary structure folding. At the sequence-level and secondary structure-level evaluations, R3Design significantly outperforms protein design baselines and secondary structure-based RNA design models. Namely, it effectively extracts RNA structural features through an RNA-specific modeling approach and introduces secondary structure constraints to help refine the designed sequence. R3Design also shows more stable performance than secondary structure-based RNA design models since most models are unable to output a conserved sequence for a large portion of RNA structure inputs in our dataset, as shown in Fig. 2. At the tertiary structure level, R3Design proves its capability to design RNA sequences that accurately fold into the desired tertiary structures, as validated by three advanced structure prediction models. Despite the designed sequences differing significantly from their native sequences, the resulting structures exhibit high similarity to the target structures. The comprehensive evaluations across multiple datasets underscore the robustness and generalizability of R3Design, establishing it as a powerful tool for RNA sequence design. The standalone R3Design software not only extends its utility but also stands out as the first comprehensive tool to tackle the entire RNA inverse folding problem. The software pipeline begins by accepting an RNA tertiary structure as input. It then proceeds with sequence design via the R3Design model and concludes by predicting the tertiary structure of the designed sequence, employing RosettaFoldNA [35] for the final output. Furthermore, the software allows for rigorous testing and evaluation of the functionalities and outcomes of each module within the model. It can generate secondary structures from the input tertiary structure in dot-bracket notation, assess the accuracy of the designed sequences, and compare the structural similarity between the input and the output predicted RNA tertiary structures. As a multifunctional tool, R3Design addresses each phase of the RNA inverse folding process. It not only promotes the development of similar in-silico models but also provides critical insights for experimental validation.

One limitation of R3Design is that it was trained using RNA sequences shorter than 500 nucleotides to enhance computational efficiency. However, as detailed in Supplementary Section A, a substantial 95.79% of sequences within the representative RNA chains are below this 500-nucleotide threshold, with longer sequences sporadically ranging between 500 and 4000 nucleotides. This distribution ensures that R3Design is applicable to the vast majority of RNA structures encountered in current databases, though its utility for exceptionally long RNA molecules remains constrained.

Another significant limitation is R3Design’s current inability to account for interactions between RNA and other molecules, such as proteins or small molecular ligands. This consideration is critical for the design of RNA molecules that function within complex biological systems, such as riboswitches or RNA aptamers that specifically bind to target molecules. Future iterations of R3Design could integrate these molecular interactions, employing more complex modeling frameworks that simulate the intermolecular forces and binding dynamics involved in these systems.

## Methods

### Preliminaries

For an RNA molecule, its primary structure consists of a sequence of nucleotide bases, which can be succinctly described by the following:


(1) \begin{align*}\mathrm{Nucleotides} &:= \{\text{A (Adenine), U (Uracil), C (Cytosine), G (Guanine)}\}, \nonumber\\ \mathcal{S}^{N} &= \{s_{i} \in \mathrm{Nucleotides} \; | \; i \in [1, N] \cap \mathbb{Z}\}, \end{align*}

where $N$ represents the total number of nucleotides in the RNA sequence. The formation of the tertiary structure involves the three-dimensional folding of this sequence, which involves specific atomic positions and can be denoted as


(2) \begin{align*}& \begin{aligned} \mathrm{Atoms} &:= \{\mathrm{P}, \mathrm{O}5^{\prime}, \mathrm{C}5^{\prime}, \mathrm{C}4^{\prime}, \mathrm{C}3^{\prime}, \mathrm{O}3^{\prime}\}, \\ \mathcal{X}^{N} &= \{\boldsymbol{x}_{i}^{\omega}\in\mathbb{R}^{3} \; \big| \; i \in [1, N] \cap \mathbb{Z}, \omega \in \mathrm{Atoms}\}, \end{aligned}\end{align*}

where $\mathrm{Atoms}$ specifies the six atoms that typically comprise the backbone of the RNA structure, necessary for the integrity of its three-dimensional form.

Additionally, the secondary structures are incorporated using the dot-bracket notation, which efficiently marks paired and unpaired nucleotides:


(3) \begin{align*}& \begin{aligned} \mathcal{A}^{N} = \{a_{i} \in \{\;.\;,\; (\;,\; )\} \; \big| \; i \in [1, N] \cap \mathbb{Z}\}, \end{aligned}\end{align*}

where $a_{i}$ is ’.’ if the nucleotide at position $i$ is unpaired, and ’(’ or ’)’ if it is part of a base pair. This notation is particularly useful in identifying and categorizing structural motifs that are crucial for the RNA’s function.

The core challenge of tertiary structure-based RNA sequence design is to formulate the mapping from the tertiary structure back to a corresponding primary structure, ideally preserving functionally important elements:


(4) \begin{align*}& \begin{aligned} \mathcal{F}_\Theta&: \mathcal{X}^{N} \mapsto \mathcal{S}^{N}, \end{aligned}\end{align*}

where $\mathcal{F}_\Theta $ represents a learnable mapping function parameterized by $\Theta $. This function essentially aims to predict the primary RNA sequence ($\mathcal{S}^{N}$) that is capable of folding into a given tertiary structure ($\mathcal{X}^{N}$).

### Graph-based RNA tertiary structure modeling

In this study, we develop a local coordinate system for each nucleotide in the RNA’s tertiary structure to facilitate precise structural modeling. The local coordinate system, denoted as $\boldsymbol{Q}_{i}$ for the $i$th nucleotide, is constructed as follows:


(5) \begin{align*}& \boldsymbol{Q}_{i} = [\mathbf{b}_{i}, \mathbf{n}_{i}, \mathbf{b}_{i} \times \mathbf{n}_{i}],\end{align*}

where $\mathbf{b}_{i}$ is the negative bisector of angles between the rays of contiguous coordinates $(\boldsymbol{x}_{i-1, \mathrm{P}}, \boldsymbol{x}_{i, \mathrm{P}})$ and $(\boldsymbol{x}_{i+1, \mathrm{P}}, \boldsymbol{x}_{i, \mathrm{P}})$, and $\mathbf{n}_{i}$ is a unit vector normal to that plane. Formally, $\mathbf{b}_{i}$ and $\mathbf{n}_{i}$ are


(6) \begin{align*}& \mathbf{u}_{i} = \frac{\mathbf{x}_{i} - \mathbf{x}_{i-1}}{\|\mathbf{x}_{i} - \mathbf{x}_{i-1}\|}, \mathbf{b}_{i} = \frac{\mathbf{u}_{i} - \mathbf{u}_{i+1}}{\|\mathbf{u}_{i} - \mathbf{u}_{i+1}\|}, \mathbf{n}_{i} = \frac{\mathbf{u}_{i} \times \mathbf{u}_{i+1}}{\|\mathbf{u}_{i} \times \mathbf{u}_{i+1}\|}.\end{align*}

Constructing the local coordinate system involves calculating the bond vectors between successive $\mathrm{P}$ atoms and using these to determine the bisector $\mathbf{b}_{i}$ and normal $\mathbf{n}_{i}$. This local coordinate system is crucial as it provides a stable frame of reference for each nucleotide, invariant to the overall rotations and translations of the RNA molecule, thereby allowing for consistent intra- and inter-nucleotide measurements.

Unlike proteins where the backbone geometry can often be sufficiently modeled using only the $\mathrm{C}\alpha $ atoms, RNA molecules exhibit a diversity of backbone conformations and base-pairing interactions that are more complex. To effectively capture this complexity, we propose modeling the RNA tertiary structure as an attributed graph $\mathcal{G} = (V, E)$, where $V$ and $E$ represent the node and edge attributes, respectively:


(7) \begin{align*}& \begin{aligned} V \in \mathbb{R}^{N \times f_{n}}, E \in \mathbb{R}^{N \times K \times f_{m}}, \end{aligned}\end{align*}

with each node $i$ connected to $K$ nearest neighbors in three-dimensional space, forming a set denoted by $\mathcal{N}(i, K)$. Here, $f_{n}$ and $f_{m}$ represent the dimensionalities of the node and edge attribute vectors, respectively. By default, we select $K=30$ to balance computational efficiency with structural detail capture.

We outline the attributes used in our modeling approach along with their corresponding illustrations in Table 4, which includes two levels of attributes: (i) intra-nucleotide level attributes describing the local geometry of each nucleotide as the node attribute $V$, and (ii) inter-nucleotide level attributes describing the relative geometry between nucleotides as the edge attribute $E$.

**Table 4:** The feature construction of RNA tertiary structure modeling.

Level	Feature	Illustration
Intra	Dihedral Angle	$\big \{\sin , \cos \big \}\times \big \{\alpha _{i}, \beta _{i}, \gamma _{i}, \delta _{i},\epsilon _{i}, \zeta _{i} \big \}$
	Distance	$\left \{\mathrm{RBF}(\|\omega _{i} - \mathrm{P}_{i}\|) \; \Big | \; \omega \in \{{\mathrm{O}5^{\prime}, \mathrm{C}5^{\prime}, \mathrm{C}4^{\prime}, \mathrm{C}3^{\prime}, \mathrm{O}3^{\prime}}\} \right \}$
	Direction	$\left \{\boldsymbol{Q}_{i}^{T} \; \frac{\omega _{i} - \mathrm{P}_{i}}{\| \omega _{i} - \mathrm{P}_{i} \|} \; \Big | \; \omega \in \{{\mathrm{O}5^{\prime}, \mathrm{C}5^{\prime}, \mathrm{C}4^{\prime}, \mathrm{C}3^{\prime}, \mathrm{O}3^{\prime}}\} \right \}$
Inter	Orientation	$\boldsymbol{q}(\boldsymbol{Q}_{i}^{T} \boldsymbol{Q}_{j})$
	Distance	$\left \{\mathrm{RBF}(\| \omega _{j} - \mathrm{P}_{i} \|) \; \Big | \; j \in \mathcal{N}(i, K), \omega \in \{{\mathrm{O}5^{\prime}, \mathrm{C}5^{\prime}, \mathrm{C}4^{\prime}, \mathrm{C}3^{\prime}, \mathrm{O}3^{\prime}}\} \right \}$
	Direction	$\Big \{\boldsymbol{Q}_{i}^{T} \; \frac{\omega _{j} - \mathrm{P}_{i}}{\| \omega _{j} - \mathrm{P}_{i}\|} \; \Big | \; j \in \mathcal{N}(i, K), \omega \in \{{\mathrm{O}5^{\prime}, \mathrm{C}5^{\prime}, \mathrm{C}4^{\prime}, \mathrm{C}3^{\prime}, \mathrm{O}3^{\prime}}\} \Big \}$

**Intra-nucleotide level**

**Figure 5:**
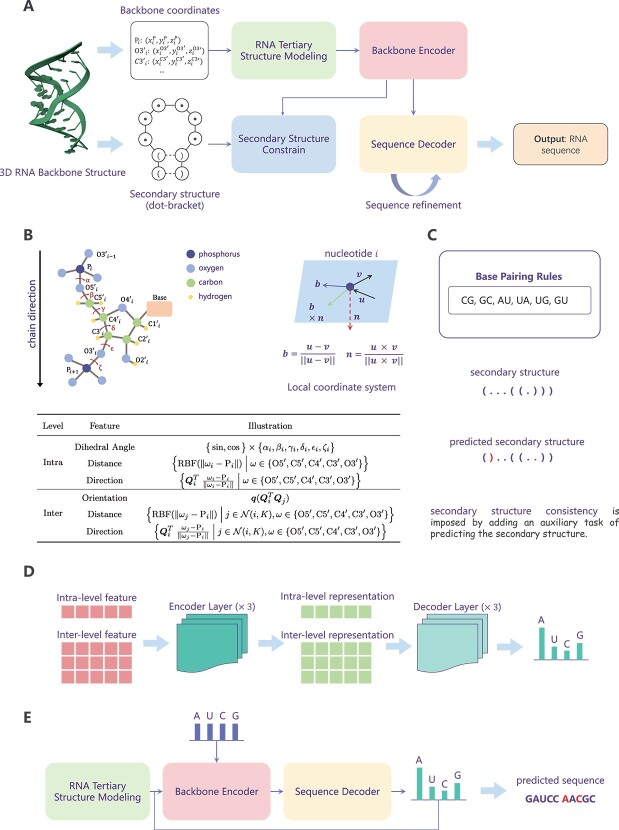
Overall framework of R3Design. (A) The overview of the R3Design pipeline. (B) The graph-based RNA tertiary structure modeling. (C) The secondary structure auxiliary task. (D) The model architecture of the backbone encoder and the sequence decoder. (E) The iterative sequence refinement process.

(1) The dihedral angles, shown as red arrows in Fig. 5B, are calculated. We represent the dihedral angles of the RNA backbone using $\sin $ and $\cos $ functions. (2) The spatial distances between *the other intra-nucleotide atoms* and the atom $\mathrm{P}_{i}$ are encoded into radial basis functions (RBFs). (3) The directions of the other intra-nucleotide atoms relative to the atom $\mathrm{P}_{i}$ are calculated with respect to the local coordinate system $\boldsymbol{Q}_{i}$.

**Inter-nucleotide level**

(1) An orientation encoding $\boldsymbol{q}(\cdot )$ is calculated from the quaternion representation of the spatial rotation matrix $\boldsymbol{Q}_{i}^{T} \boldsymbol{Q}_{j}$. (2) The spatial distances between inter-nucleotide atoms from *neighboring nucleotides* and the atom $\mathrm{P}_{i}$ are encoded into RBFs. (3) The directions of the other inter-nucleotide atoms relative to the atom $\mathrm{P}_{i}$ are calculated.

### The R3Design framework

With the graph-based RNA tertiary structure modeling in place, we introduce the R3Design framework as shown in Fig. 5, which is structured around two principal components: a backbone encoder and a sequence decoder.

The backbone encoder is designed to transform the complex RNA tertiary structure into a comprehensive latent representation. It utilizes three layers of PiGNN, an adaptation from the PiFold protein design model [44]. This encoder processes the graph representation of the RNA tertiary structure meticulously, capturing not just the structural intricacies but also the crucial spatial relationships between nucleotides. Through its multilayered architecture, the backbone encoder effectively distills the essential features from the tertiary structure into a condensed form, setting the stage for accurate sequence prediction.

The sequence decoder is tasked with designing the corresponding RNA sequence from the latent representation provided by the encoder. The sequence decoder employs a linear layer optimized for this purpose, ensuring that the transition from structural data to nucleotide sequence is both smooth and accurate. This layer is intricately conditioned on the latent representation of the RNA tertiary structure, ensuring that structural information captured by the backbone encoder is effectively translated into the designed RNA sequence.

With the graph-based RNA tertiary structure modeling in place, we introduce the R3Design framework, which comprises two primary components: a backbone encoder and a sequence decoder. The backbone encoder is responsible for encoding the RNA tertiary structure into a latent representation, while the sequence decoder generates the corresponding RNA sequence from this representation. The backbone encoder is implemented using three layers of PiGNN [44], which is adapted from the protein design model PiFold. It processes the graph representation of the RNA tertiary structure, capturing the structural intricacies and spatial relationships between nucleotides. The sequence decoder, on the other hand, is a linear layer that predicts the RNA sequence based on the learned representation. It is conditioned on the latent representation of the RNA tertiary structure.

**Secondary structure constraint**

To ensure that the learned representations robustly encapsulate the functional intricacies of RNA sequences, particularly with respect to their secondary structures, we have incorporated a secondary structure constraint into our model. This constraint is operationalized through an auxiliary task that focuses on predicting the RNA’s secondary structure in the dot-bracket notation, which serves as a critical intermediary step in understanding RNA folding patterns. As shown in Fig. 5C, The auxiliary prediction task involves the use of the dot-bracket notation, a conventional method for denoting the secondary structure of RNA molecules. In this format, unpaired nucleotides are represented by dots (’.’), and paired nucleotides are bracketed together with matching parentheses (’(’ and ’)’), indicating base pairs in the RNA structure. This task is integrated into the main learning process to ensure that the secondary structural features are effectively captured in the RNA representation model. We implement a Transformer layer [45] to predict the secondary structure by giving the latent representation of the RNA tertiary structure. This Transformer layer is trained to predict the secondary structure of the RNA molecule, ensuring that the model captures the essential structural features of the RNA sequence. The secondary structure constraint is employed by the cross-entropy loss between the predicted secondary structure and the ground-truth secondary structure:


(8) \begin{align*}& \mathcal{L}_{sec} = - \sum_{i=1}^{N} \sum_{j\in\{\mathrm{., (, )}\}} a_{i,j}^{*} \log a_{i,j},\end{align*}

where $a_{i,j}^{*}$ and $a_{i,j}$ are the ground-truth and predicted probabilities of the $j$th secondary structure type at the $i$th position, respectively.

**Iterative sequence refinement**

We initialize the model with a uniform probability distribution across the four nucleotide types (Adenine, Uracil, Cytosine, Guanine) for each position in the nucleotide sequence. Formally, this initialization can be described as follows:


(9) \begin{align*}& \mathcal{P}_{0} = \{p_{i,j}=0.25 \;|\; i \in [1, N] \cap \mathbb{Z}, j \in \mathrm{\{A,U,C,G}\} \}\,\end{align*}

where $\mathcal{P}_{0}$ represents the initial probability distribution, $N$ is the length of the RNA sequence, and $p _{i,j}$ is the probability of the $j$th nucleotide type at the $i$th position, with each type having an equal probability of $0.25$ initially.

The input to the model consists of this distribution of nucleotides, $\mathcal{P}_{0}$, combined with the graph-based representation of the RNA’s tertiary structure, $\mathcal{G}$. The model is trained to adjust its parameters to predict the probability distribution of the nucleotide sequence that aligns with the actual biological sequence observed. The predicted distribution of the $k$-iteration, $\mathcal{P}_{k}$, is refined through iterative training to closely approximate the ground-truth distribution, $\mathcal{P}*$. The training objective is to minimize the cross-entropy loss between the predicted probability distributions and the ground-truth distribution across all $K$ iterations:


(10) \begin{align*}& \mathcal{L}_{\textrm{sup}} = - \sum_{k=1}^{K} \sum_{i=1}^{N} \sum_{j\in\{\mathrm{A,U,C,G}\}} p_{i,j}^{*} \log p^{k}_{i,j},\end{align*}

where $p_{i,j}^{*}$ and $p^{k}_{i,j}$ are the ground-truth and predicted probabilities of the $j$th nucleotide type at the $i$th position at the $k$-iteration, respectively. We set the number of iterations $K=3$ in default.

The overall training objective is the linear combination of the secondary structure constraint loss and the iterative sequence loss:


(11) \begin{align*}& \mathcal{L} = \mathcal{L}_{sup} + \mathcal{L}_{sec}.\end{align*}

The architecture of the R3Design model is depicted in Fig. 5D, which illustrates the integration of the backbone encoder, sequence decoder, and iterative refinement.

**Key Points**

The main messages we would like to express in the paper are listed as follows:

- This work introduces R3Design, a method designed to design RNA sequences based on their tertiary structures, trained on over two thousand RNA structures from the Protein Data Bank (PDB).
- The method uses base pair prediction to improve accuracy by connecting RNA secondary and tertiary structures.
- R3Design uses an iterative refinement process, which adjusts its predictions over multiple cycles to better match the complex structure of RNA.
- This method is integrated into standalone software with another structure prediction approach, providing a comprehensive toolkit for designing, folding, and evaluating RNA at the tertiary level.

## Supplementary Material

appendices_bbae682_bbae682

## Data Availability

The benchmark data used by this paper is publicly available at https://github.com/A4Bio/R3Design/releases/tag/data. Rfam and RNA-Puzzles datasets are publicly available at https://zhanggroup.org/DeepFoldRNA/benchmark.html.

## Code availability

The R3Design software package, implemented by PyTorch, is publicly available at https://github.com/A4Bio/R3Design.
